# Supplementary material for: Sensory Evaluation of Common Ice Plant (Mesembryanthemum crystallinum L.) in Response to Sodium Chloride Concentration in Hydroponic Nutrient Solution
Source: Foods. 2022 Sep 9;11(18):2790. doi: 10.3390/foods11182790 (PMC9497535; doi:10.3390/foods11182790)
Supplement: Supplementary file 1 [file foods-11-02790-s001.zip › foods-1829337-supplementary.pdf]

Table S1. Questionnaire

**Repeated for all test samples…**	
**Appearance**	
How would you rate this test sample's overall APPEARANCE?	9=Like it extremely, 8=Like it very much, 7=Like it moderately, 6=Like it slightly, 5=Neither like nor dislike it, 4=Dislike it slightly, 3=Dislike it moderately, 2=Dislike it very much, 1=Dislike it extremely
Describe the appearance of this product (open-ended)	Word description
Which COLOR from the given sheet matches the sample?	1-9 color swatches
Which color from the given sheet would be IDEAL for this plant	1-9 color swatches
**Overall liking**	
Please rate your OVERALL liking of this product	9=Like it extremely, 8=Like it very much, 7=Like it moderately, 6=Like it slightly, 5=Neither like nor dislike it, 4=Dislike it slightly, 3=Dislike it moderately, 2=Dislike it very much, 1=Dislike it extremely
What do you LIKE or DISLIKE about this product?	Word description
**Flavor**	
How would you DESCRIBE the FLAVOR of this product?	Word description
Rate the overall flavor of this product	9=Like it extremely, 8=Like it very much, 7=Like it moderately, 6=Like it slightly, 5=Neither like nor dislike it, 4=Dislike it slightly, 3=Dislike it moderately, 2=Dislike it very much, 1=Dislike it extremely
How strong is the saltiness for the product	7=very strong…1=not at all
Think about SALTINESS, would you say it was ...	5=much too salty, 4=somewhat too salty, 3=just about right, 2=not quite salty enough, 1=not at all salty enough
How strong is the bitterness?	7=very strong…1=not at all
How strong is the sweetness?	7=very strong…1=not at all
How strong is the sourness?	7=very strong…1=not at all
How strong is the green flavor?	7=very strong…1=not at all
How strong is the fishiness for the product	7=very strong…1=not at all
**Texture**	
How would you rate the overall TEXTURE of this sample?	9=Like it extremely, 8=Like it very much, 7=Like it moderately, 6=Like it slightly, 5=Neither like nor dislike it, 4=Dislike it slightly, 3=Dislike it moderately, 2=Dislike it very much, 1=Dislike it extremely
Think about CRUNCHINESS, would you say it was ...	5=much too crunchy, 4=slightly too crunchy, 3=just about right, 2=not quite crunchy enough, 1=not at all crunchy enough
Think about JUICINESS, would you say it was ...	5=much too juicy, 4=slightly too juicy, 3=just about right, 2=not quite juicy enough, 1=not at all juicy enough
Think about TOUGHNESS, would you say it was ...	5=much too tough, 4=slightly too tough, 3=just about right, 2=not quite tough enough, 1=not at all tough enough
**Aftertaste**	
Do you notice any aftertaste	Yes/No
If "yes", Please describe the aftertaste you perceived	Word description
If "yes", is the aftertaste acceptable	Yes/No
**Asked once all the samples have been evaluated…**	

**Familiarity**	
Thinking about ICE PLANT, would you say you are...?	1=not at all familiar, 2=not too familiar, 3=slightly familiar, 4=moderately familiar, 5=very familiary
**Purchase Intent**	
Ice plant is a succulent perennial plant with edible leaves, served in the upscale restaurants. The researchers tried to popularized this plant and would like to know...	
How likely would you be to purchase this product if it was available at a reasonable price where you normally shop?	1=definitely would not purchase, 2=probably would not purchase, 3=may or may not purchase, 4=probably would purchase, 5=definitely would purchase
**Comparison to salad greens**	
Would you find this appealing compared with regular salad greens?	1=much less appealing, 2=somewhat less appealing, 3=same, 4=somewhat more appealing, 5=much more appealing than salad greens
**Variety-/Sensation-Seeking Behaviors**	
Please indicate which of the following scenarios you would prefer.	1=I like to try new foods that I have never tasted before<br>2=I order the dishes with which I am familiar so as to avoid disappointment and unpleasantness
Sensation Seekers are willing to try out new food, including novel, unusual, or exotic foods…<br>How much do you agree or disagree with this statement?<br>Do you think you are one of the sensation seeker?	1=strongly disagree…7=strongly agree
**Ice Plant consumption and preparation**	
In what OCCASION or SITUATION do you find yourselves eating this plant?	Open-ended
If you are going to prepare ice plant for cooking? How would you prepare and serve the dish?	Open-ended
**Salt consumption**	
Do you worry about salt consumption?	1=no, 2=yes
The samples you tasted had much higher salt level than Lay's classic potato chips, would that be a concern?	1=definitely not a concern, 2=probably not a concern, 3=may or may not be a concern, 4= probably is a concern, 5=definitely is a concern
**Demographics**	
Please specify your gender.	1=male, 2= female
Please specify your age	Open-ended
Which of the following best describes your ethnic background?	1=White/Caucasian, 2=Hispanic/Latino, 3=Black/African American, 4=Native American/American Indian, 5=Asian/Pacific Islander, 6=other

Table S2. Principal Component Analysis details: a) Eigenvalues, b) Factor Loadings (green = highly positively associated with a factor, dark red = highly negatively associated with a factor), c) Scree Plot.

a)

	F1	F2	F3	F4
Eigenvalue	11.374	2.094	1.752	0.780
Variability (%)	71.087	13.088	10.950	4.876
Cumulative %	71.087	84.174	95.124	100.000

c)

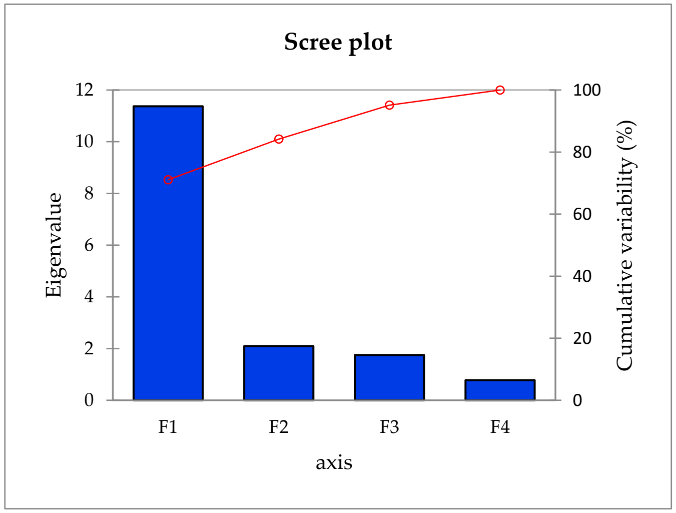

b)

	F1	F2	F3	F4
Overall Liking	-0.988	-0.012	0.138	0.073
Appearance Liking	0.270	0.815	-0.509	0.062
Color assessment	0.959	-0.027	0.275	0.057
Flavor Liking	-0.979	0.179	0.084	0.046
Salty intensity	0.993	-0.066	-0.092	0.036
Salty JAR	0.988	-0.090	-0.107	0.057
Bitterness	0.633	0.587	0.107	-0.493
Sweetness intensity	-0.957	0.249	0.130	0.077
Sourness intensity	1.000	-0.005	0.018	0.025
Green flavor	-0.958	0.044	-0.279	0.045
Fishy flavor	0.964	-0.010	-0.264	-0.020
Texture Liking	0.297	0.605	0.737	-0.052
Crunchiness	0.784	-0.616	-0.070	-0.010
Juiciness	-0.776	0.128	-0.618	0.008
Toughness	0.594	0.353	0.131	0.711
notice any aftertaste?	0.813	0.298	-0.499	-0.021

Table S3. Agglomerative Hierarchical Cluster Analysis (AHC) details: a) Variance decomposition for the optimal classification, b) Dendrogram, c) Results by class, d) Class membership(%) vs. NaCl concentration; e) Class membership (%) vs. Total study population (n=115); f) ANOVA (Observation by Class membership), small letters indicate statistically significant difference at 95% Level of Confidence based on Tukey's HSD Test.

a)

	Absolute	Percent
Within-class	27.689	76.33%
Between-classes	8.586	23.67%
Total	36.276	100.00%

b) Dendrogram

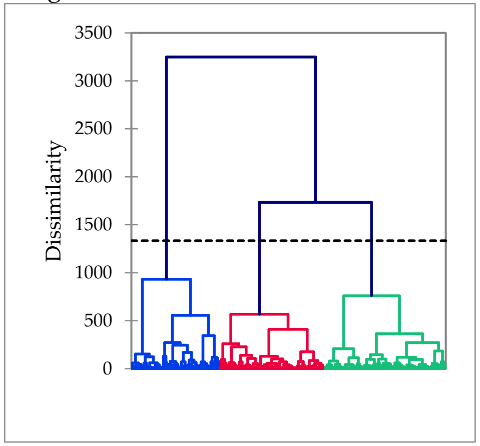

c)

Class	1	2	3
Objects	221	192	162
Sum of weights	221	192	162
Within-class variance	23.677	24.727	36.685
Minimum distance to centroid	1.414	2.491	2.279
Average distance to centroid	4.678	4.783	5.863
Maximum distance to centroid	9.546	9.274	10.562

d)

Class	0.00M NaCl	0.05M NaCl	0.10M NaCl	0.20M NaCl	0.40M NaCl	% Total Observations
1	49	39	37	34	33	38
2	44	48	40	25	10	33
3	7	13	23	41	57	28

e)

Class	% Study Population (total n=115)
1	83
2	70
3	72

f)

	1	2	3	Pr > F(Model)	Pr > F(Cluster)
Overall Liking (9pt)	6.484 a	4.443 b	3.025 c	< 0.0001	< 0.0001
Appearance Liking (9pt)	6.887 a	5.573 b	5.778 b	< 0.0001	< 0.0001
Color assessment (7pt)	3.615 a	3.042 b	3.716 a	< 0.0001	< 0.0001
Flavor Liking(9pt)	6.471 a	4.484 b	2.840 c	< 0.0001	< 0.0001
Salty intensity(7pt)	3.376 b	2.651 c	5.407 a	< 0.0001	< 0.0001
Bitter intensity(7pt)	2.222 b	1.927 b	2.710 a	< 0.0001	< 0.0001
Sweetness intensity(7pt)	1.919 a	1.396 b	1.519 b	< 0.0001	< 0.0001
Sourness intensity(7pt)	2.127 b	1.333 c	2.562 a	< 0.0001	< 0.0001
Green flavor intensity(7pt)	3.851 a	3.229 b	3.241 b	< 0.0001	< 0.0001
Fishy flavor intensity(7pt)	2.063 b	1.391 c	3.938 a	< 0.0001	< 0.0001
Texture Liking(9pt)	6.878 a	4.906 b	5.278 b	< 0.0001	< 0.0001
